# Profiling structured product labeling with NDF-RT and RxNorm

**DOI:** 10.1186/2041-1480-3-16

**Published:** 2012-12-20

**Authors:** Qian Zhu, Guoqian Jiang, Christopher G Chute

**Affiliations:** 1 Department of Health Sciences Research, Division of Biomedical Statistics and Informatics, Mayo Clinic, Rochester, MN, USA

## Abstract

**Background:**

Structured Product Labeling (SPL) is a document markup standard approved by Health Level Seven (HL7) and adopted by United States Food and Drug Administration (FDA) as a mechanism for exchanging drug product information. The SPL drug labels contain rich information about FDA approved clinical drugs. However, the lack of linkage to standard drug ontologies hinders their meaningful use. NDF-RT (National Drug File Reference Terminology) and NLM RxNorm as standard drug ontology were used to standardize and profile the product labels.

**Methods:**

In this paper, we present a framework that intends to map SPL drug labels with existing drug ontologies: NDF-RT and RxNorm. We also applied existing categorical annotations from the drug ontologies to classify SPL drug labels into corresponding classes. We established the classification and relevant linkage for SPL drug labels using the following three approaches. First, we retrieved NDF-RT categorical information from the External Pharmacologic Class (EPC) indexing SPLs. Second, we used the RxNorm and NDF-RT mappings to classify and link SPLs with NDF-RT categories. Third, we profiled SPLs using RxNorm term type information. In the implementation process, we employed a Semantic Web technology framework, in which we stored the data sets from NDF-RT and SPLs into a RDF triple store, and executed SPARQL queries to retrieve data from customized SPARQL endpoints. Meanwhile, we imported RxNorm data into MySQL relational database.

**Results:**

In total, 96.0% SPL drug labels were mapped with NDF-RT categories whereas 97.0% SPL drug labels are linked to RxNorm codes. We found that the majority of SPL drug labels are mapped to chemical ingredient concepts in both drug ontologies whereas a relatively small portion of SPL drug labels are mapped to clinical drug concepts.

**Conclusions:**

The profiling outcomes produced by this study would provide useful insights on meaningful use of FDA SPL drug labels in clinical applications through standard drug ontologies such as NDF-RT and RxNorm.

## Introduction

Structured Product Labeling (SPL) [1] encodes very rich clinical drug knowledge, such as dosage, strength, usage of drug, etc. The importance of this resource is widely recognized, and it has been utilized in multiple studies [2,3] to support clinical and translational research use cases. For example, the relationships between genes, diseases, drugs, and adverse events available from SPL drug lavels can assist clinicians to improve the safety and effectiveness of treatments, and help translational researchers to design novel bioinformatics algorithms. However, the drug information/knowledge written into SPL drug labels is currently in unstructured free text instead of structured codified information, which poses significant challenges to computational analysis of the knowledge, and hinders the integration of SPL drug labels with other existing knowledge bases. Actually, this is a common scenario occurring in the biomedical domain, where dozens of public resources involve laborious processes to manually annotate data. This is mostly because they are using heterogeneous code systems to represent their data. Hence, data normalization and building all possible linkages among these data sets will make data interoperation and integration feasible.

Semantic Web Technology (SWT) [4] can be useful to provide a scalable framework for facilitating semantic data integration of heterogeneous resources and enabling semantic sharing through the standard query services. It has been widely used in biomedical domains to formalize and model medical and biological systems [5-7]. In the present study, we adopted it as the core technology in our implementation step.

The objective of the present study is to map SPL drug labels into two major standard drug ontologies: the Veterans Administration’s (VA) National Drug File Reference Terminology (NDF-RT) [8] and the National Library of Medicine’s (NLM) RxNorm [9]. Our investigation was guided by answering the following research questions: (1) how SPL drug labels are covered and connected by RxNorm and NDF-RT; (2) how to utilize RxNorm/NDF-RT drug resources to map SPL drug labels from the drug class and clinical drug perspective; (3) how to explore the mapping results to build a drug /drug class network; (4) how to leverage Semantic Web technology to accomplish the implementation task.

The paper is organized into the following sections. First, we introduce background information for SPL, NDF-RT, RxNorm and Semantic Web technology in the Background section; Second, in the Methods section, we introduce three main parallel approaches on SPL drug label profiling; Third, we illustrate our results generated from each step in the Results section, and then followed by Discussion and Conclusion.

## Background

### Structured Product Labeling (SPL)

Structured Product Labeling (SPL) is a document markup standard approved by Health Level Seven (HL7) [10] and adopted by FDA as a mechanism for exchanging product information. SPL defines the human readable label documents that contain structured content of labeling (all text, tables and figures) for a product, along with additional machine readable information (i.e., drug listing data elements including information about the product and the packaging). SPLs for all drug products marketed in the United States are available for download from the National Library of Medicine's DailyMed website [11] and they were being used in this study.

### National Drug File Reference Terminology (NDF-RT)

NDF-RT [8] is used for modeling drug characteristics including ingredients, chemical structure, dose form, physiologic effect, mechanism of action, pharmacokinetics, and related diseases.

**Figure 1:**
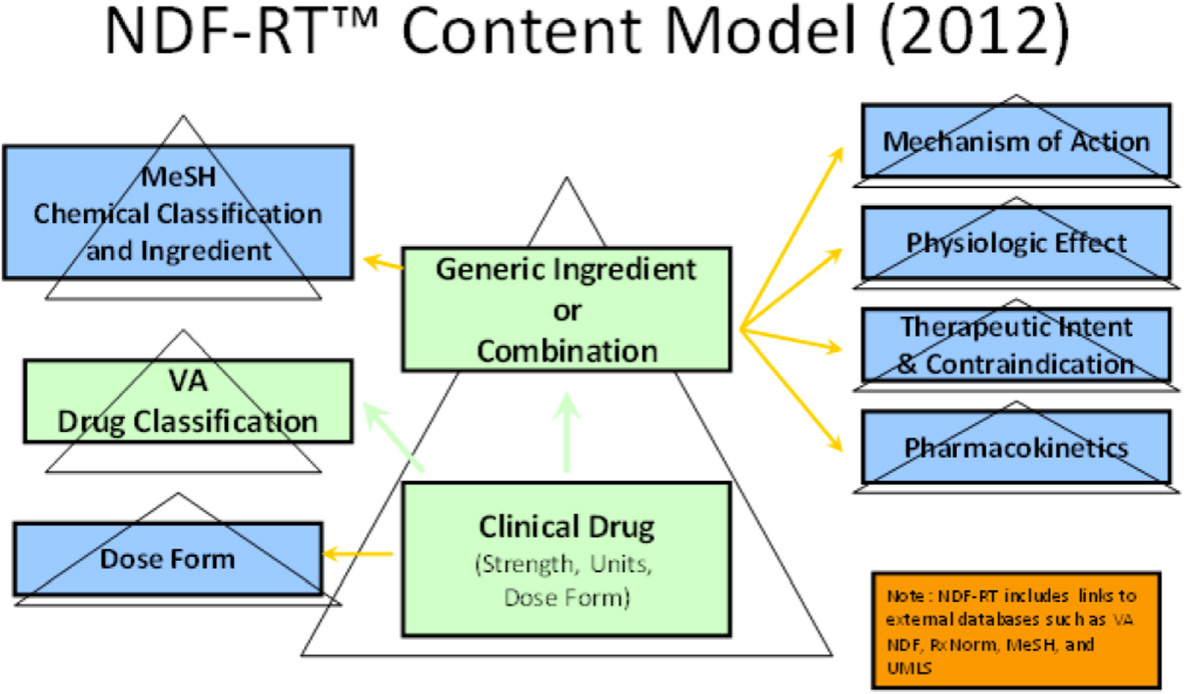
NDF-RT content model (source from U.S. Department of Veterans Affairs. NDF-RT Documentation April 2012 Version).

In support of SPL initiative, a non-hierarchical collection of External Pharmacologic Class (EPC) concepts has been added to NDF-RT in parallel and analogous with the VA Drug Classification hierarchy. These concepts are distinguished by an “[EPC]” tag suffixed to their preferred names. Role relationships describing and defining concepts according to their relationships with other concepts, originating from these EPC concepts target concepts from the NDF-RT Mechanism of Action (MoA), Physiologic Effect (PE), and Chemical Ingredient (CI) hierarchies that are selected by the FDA to index their EPC for SPL purposes [12]. The content model of NDF-RT is shown in Figure 1. Three kinds of drug concepts are involved in this study, “VA Product”, “Chemical Ingredient” and “EPC”, of which “VA Product”, and “Chemical Ingredient” are relevant to “Clinical Drug”, “Generic Ingredient or Combination” and “EPC” is analogous with the “VA Drug Classification” respectively.

### RxNorm

**Figure 2:**
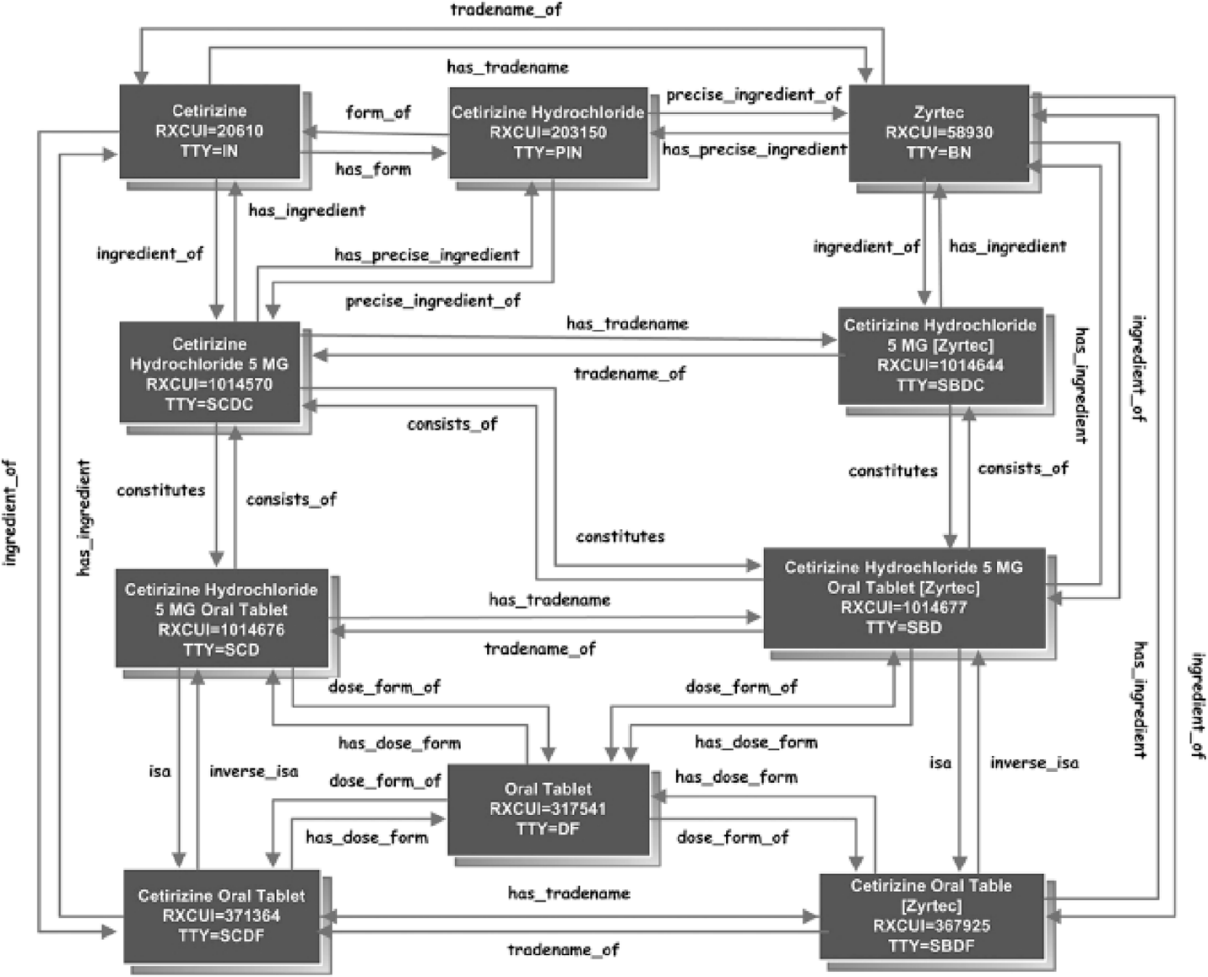
RxNorm term types relationships (Source from: Nelson SJ, Normalized names for clinical drugs: RxNorm at 6 years. JAMIA, 2011).

RxNorm [9] provides normalized names for clinical drugs and links its names to many of the drug vocabularies commonly used. RxNorm reflects and preserves the meanings, concept names, and relationships from these different copyright holders, such as SPL, NDF-RT, MeSH, and etc. The “SAB” code is defined by RxNorm to differentiate the different sources aggregated into RxNorm. For example, “MTHSPL” indicates that the corresponding concept is absorbed from SPL and “NDFRT” indicating the source from NDF-RT. These two sources were used in this study. RxNorm defines term type “TTY” to indicate the role an atom plays in its source. The term types are assigned based on source documentation or NLM understanding of the source. Table 1 shows a list of term types “TTYs” used in this study with their names and descriptions, and the relationships among these term types are shown in Figure 2.

**Table 1:** **A list of RxNorm term types “TTYs” with names and descriptions (Source from RxNorm Documentation**[13]**)**

**TTY**	**Name**	**Description**
SBD	Semantic Branded Drug	Ingredient + Strength + Dose Form + Brand Name
SCD	Semantic Clinical Drug	Ingredient + Strength + Dose Form
IN	Ingredients	A compound or moiety that gives the drug its distinctive clinical properties.
PIN	Precise Ingredient	A specified form of the ingredient that may or may not be clinically active.
BPCK	Brand Name Pack	{# (Ingredient Strength Dose Form) / # (Ingredient Strength Dose Form)} Pack [Brand Name]
GPCK	Generic Pack	{# (Ingredient + Strength + Dose Form) / # (Ingredient + Strength + Dose Form)} Pack
BN	Brand Name	A proprietary name for a family of products containing a specific active ingredient.
MIN	Multiple Ingredients	Two or more ingredients appearing together in a single drug preparation, created from SCDF.
SY	Synonym	Synonym of another TTY, given for clarity.
TMSY	Tall Man Lettering Synonym	Tall Man Lettering synonym of another TTY, given to distinguish between commonly confused drugs.

### Semantic web technology

Resource Description Framework (RDF) [14], a W3C recommendation, is a directed, labeled graph data format for representing information in the Web. SPARQL is a query language for RDF graphs [15]. RDF triple store is a database for the storage and retrieval of RDF metadata, ideally through standard SPARQL query language. Web Ontology Language (OWL) is a standard ontology language for the Semantic Web [16]. NDF-RT and EPC indexing SPL data used in this study are stored in a RDF triple store and by executing SPARQL queries to retrieve the desirable information. With the advance of SWT, linked data has been developed to describe a method of publishing structured data on the web so that it can be interlinked and become more useful.

## Materials

### SPL

**Figure 3:**
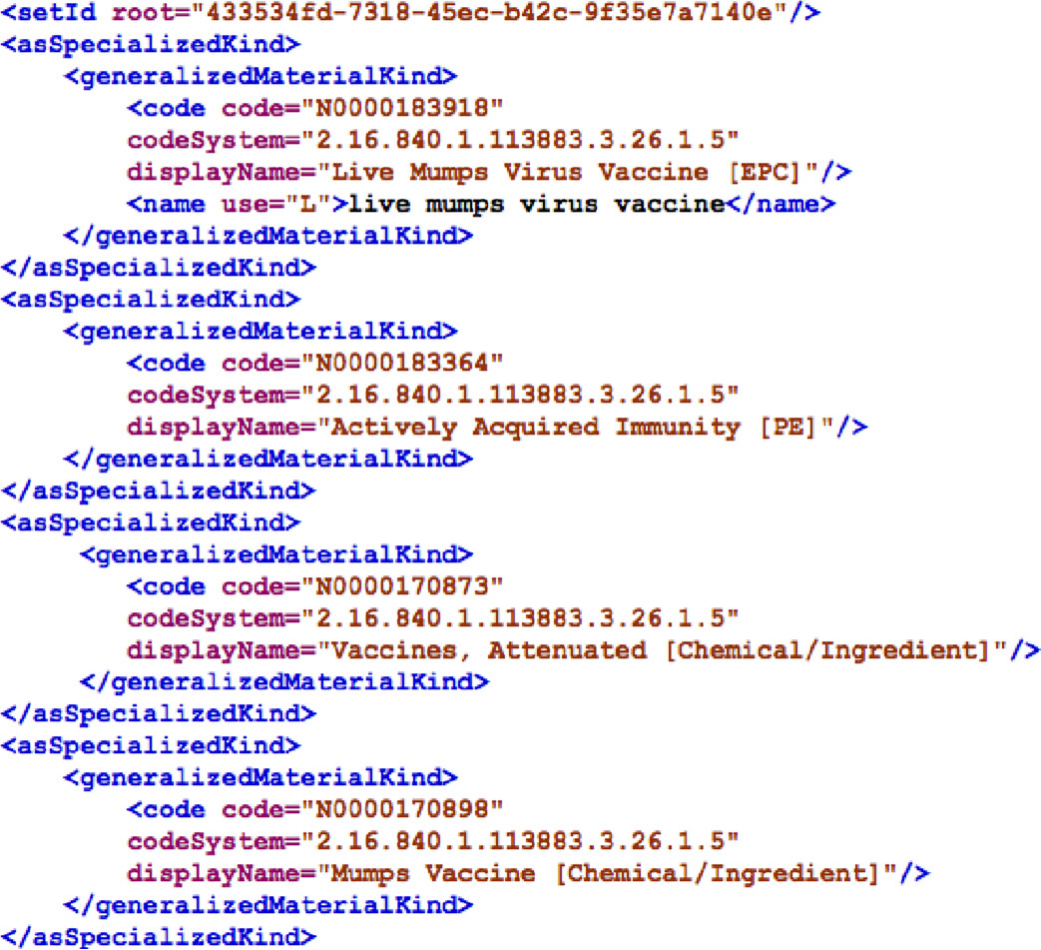
An example of EPC indexing SPL label.

In total, 1,247 EPC indexing SPLs in XML format were downloaded from NLM DailyMed website [11] as of April 12, 2012. An example of the EPC indexing SPL is shown in Figure 3. Each SPL labelled by setId (SPL unique identifier) is corresponding to one or multiple EPC classes, which mapped to NDF-RT concepts by role relationships. Totally three role relationships were identified from EPC indexing SPL files: “PE” standing for physiologic effect; “MoA” standing for mechanism of action; and “Chemical/Ingredient”.

### RxNorm

In this study, we used the following two files downloaded from RxNorm in April 9, 2012: 1) RXNCONSO.RRF. The file includes all connections (965,968 in total) with different source vocabularies. We used the data from two sources labeled as “MTHSPL” (the source from SPL) and “NDFRT” (the source from NDF-RT) in this study; 2) RXNSAT.RRF. The file includes all source vocabulary attributes that do not fit into other categories. We used the file to search for the information about drug categories and connections among RxNorm, NDF-RT and SPL. There are 6,221,513 entries included in this file. The data from both of these two files were loaded into a local MySQL database.

## Methods

### System architecture

**Figure 4:**
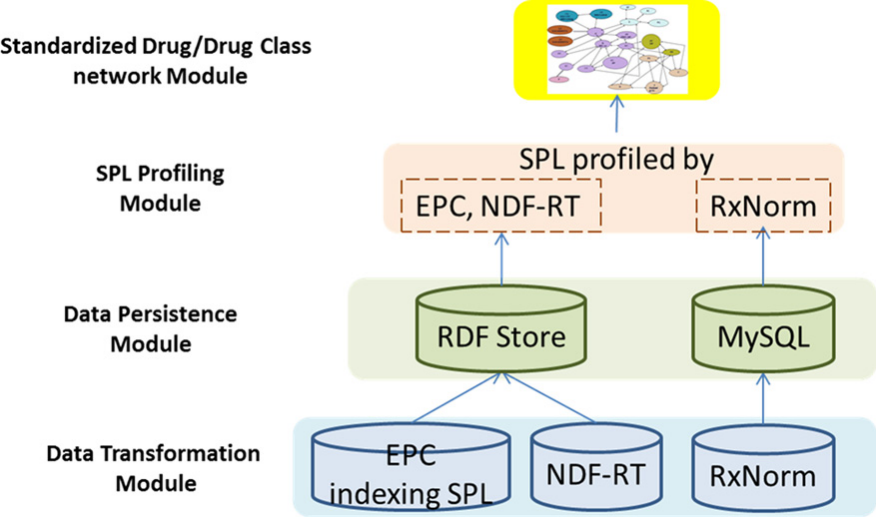
A diagram illustrating our system architecture.

There are four primary modules in the system, comprising 1) a data transformation module; 2) a data persistence module; 3) a SPL profiling module, in which SPL drug labels are profiled by EPC, NDF-RT and RxNorm; 4) a standardized drug/drug class network module. Figure 4 shows system architecture of the four modules.

For the data transformation module, data reformatting steps were performed for EPC indexing SPL, NDF-RT and RxNorm individually before loading the data into RDF tripe store and MySQL database since they are in different data formats: SPL in XML format, NDF-RT available in OWL, and RxNorm in the UMLS Rich Representation Format (RRF). A XML2RDF sub module [17] takes input rendered in the XML format, and outputs result in the RDF format through a transparent transformation service. NDF-RT in OWL was loaded into RDF store directly. RxNorm provides MySQL script for loading the data into MySQL database easily.

For the persistence module, we implemented an open source RDF store “4Store” that is developed by Garlik [18] and used the RDF store to host the SPL and NDF-RT data. After loading RDF triples into the RDF store, we implemented a SPARQL endpoint providing standard SPARQL query service against the RDF store.

For the drug/drug class network module, we incorporated SPL profiling results with our previous standardized drug work [19]. We explored to use Cytoscape [20] as a general platform for complex network analysis and visualization.

### Profiling by EPC classes

An EPC indexing SPL label is corresponding to one or multiple EPC classes and is also corresponding to one to multiple NDF-RT concepts via the following role relationships: “has_Chemical_Structure”, “has_MoA” or “has_PE”. For example, an EPC indexing SPL label, “BACILLUS CALMETTE-GUERIN SUBSTRAIN TICE LIVE ANTIGEN” is mapped to two EPC classes. The first EPC class is “Live Attenuated Bacillus Calmette-Guerin Vaccine [EPC]”, which is further mapped to “Actively Acquired Immunity [PE]”, “Vaccines, Attenuated [Chemical/Ingredient]”, and “BCG Vaccine [Chemical/Ingredient]”; and the second EPC class is “Live Attenuated Intravesical Bacillus Calmette-Guerin Vaccine [EPC]”, which is further mapped to “Increased Macrophage Proliferation [PE]”, “Increased Immunologically Active Molecule Activity [PE]”, “Vaccines, Attenuated [Chemical/Ingredient]”, and “BCG Vaccine [Chemical/Ingredient]”.

**Figure 5:**
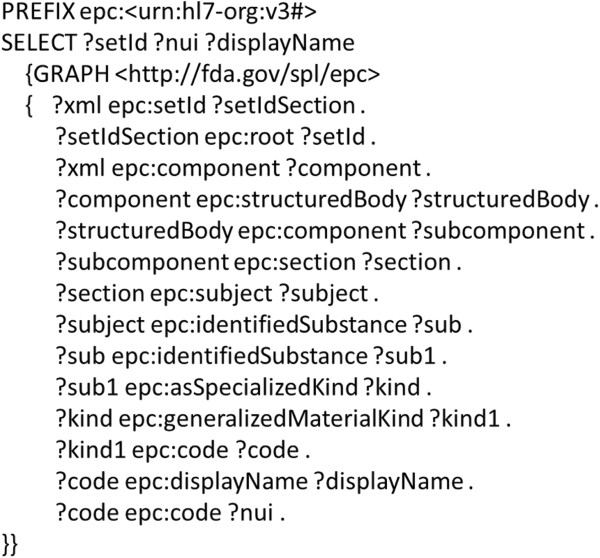
A SPARQL query for extracting EPC classes and NDF-RT concepts with their role relationships.

The EPC indexing SPL were stored in a RDF triple store. We executed a SPARQL query (as shown in Figure 5) against the triple store and extracted setId (unique identifier of SPL), NUI (unique identifier of NDF-RT), and the relevant role relationships for each EPC indexing SPL. The outcome of this query is a list of setIds and relevant NDF-RT concepts with NUIs and display names. Category information is embedded in the display name, such as “Androgen Receptor Inhibitor [EPC]” indicates “Androgen Receptor Inhibitor” is an EPC class, and “Aminoglycosides [Chemical/Ingredient]” indicates “Aminoglycosides” is a chemical ingredient.

### Profiling by RxNorm and NDF-RT

The objective of this step was to use existing annotations from RxNorm to categorize SPL drug labels, and to make connections between SPL and RxNorm/NDF-RT. As RxNorm data (RXNCONSO and RXNSAT) were pre-loaded in a MySQL database, we executed SQL queries to extract data from two RxNorm integrated resources: SPL and NDF-RT, which are differentiated by individual SAB labels.

**Figure 6:**
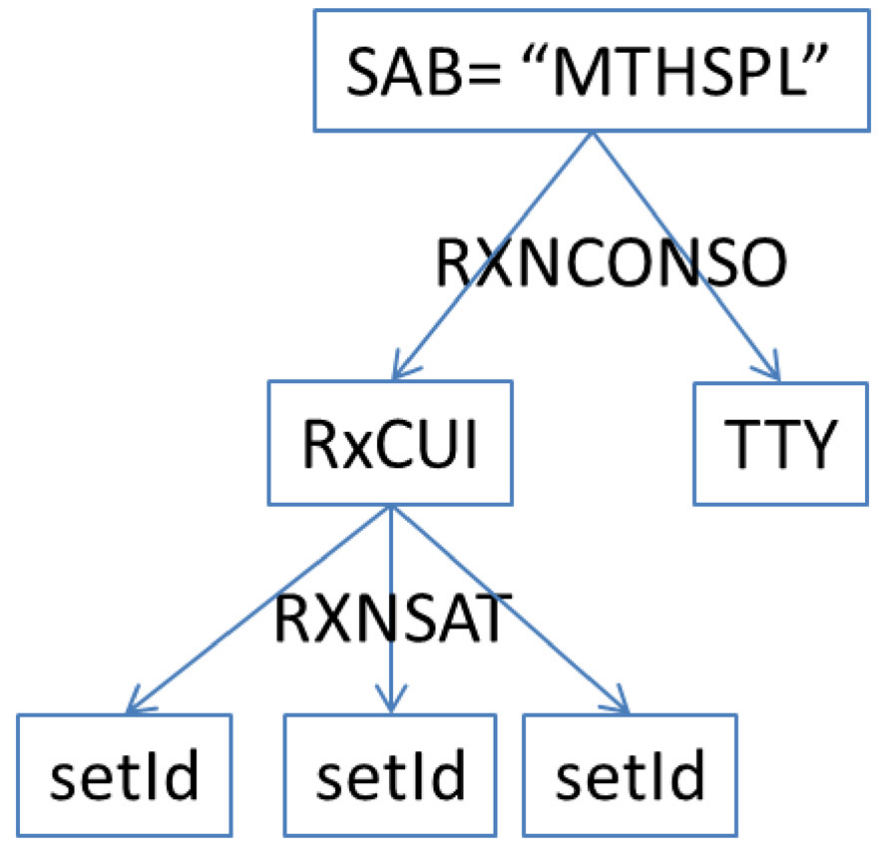
Data extraction workflow for RxNorm and SPL mappings.

For the source SPL (i.e., “SAB = MTHSPL”), we extracted concept names along with “TTY” (term type) as described in the Materials section, and RxCUIs (RxNorm unique identifier) from the RXNCONSO table. To establish linkages between SPL and RxNorm, we searched the RXNSAT table for a list of setIds with a given RxCUI. It is worthy to note that one RxCUI can correspond to multiple SPLs due to different product labellers. Figure 6 shows the workflow of the data extraction in this step.

**Figure 7:**
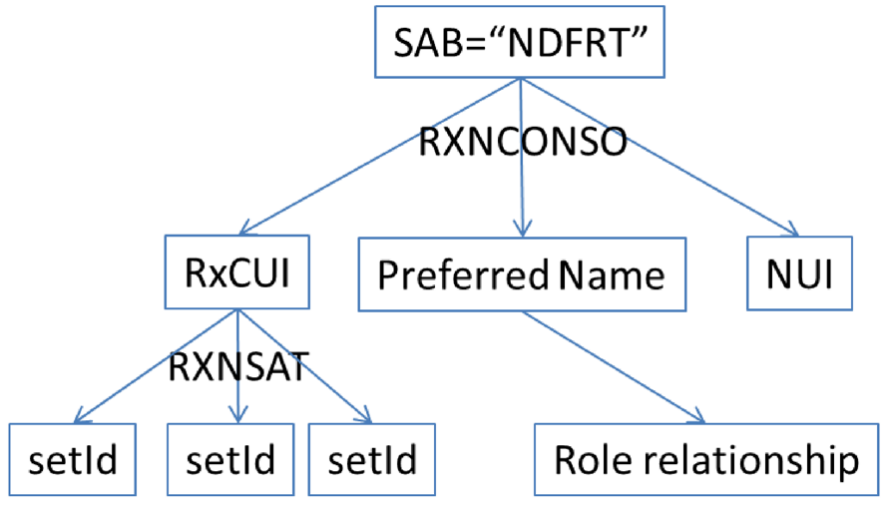
Data extraction workflow for NDF-RT and SPL mappings.

For the source NDF-RT (i.e., “SAB = NDFRT”), SQL queries were executed to extract concept names along with NUIs and preferred names. Each preferred name includes role relationships information. For example, an entry with [RxCUI = “4278”] corresponds to the [NUI = “N0000006373”] with preferred name “Famotidine [Chemical/Ingredient]”. We grouped this concept into “Chemical/Ingredient” category. Taking the same process as we did for SPL, we searched the RXNSAT table for a list of setIds with a given RxCUI and established linkages between NDF-RT and SPL. Figure 7 show the workflow of the data extraction in this step.

## Results

EPC indexing SPL, NDF-RT and RxNorm were used for profiling SPL drug labels with detailed category and standardized drug information. We presented the outcomes from each resource below.

### Results from EPC classes

The mapping results based on EPC indexing SPL labels are listed in Table 2. In total, 354 EPC unique classes were identified from the EPC indexing SPL labels, which link to 853 unique SPLs. In the meantime, we extracted all individual NDF-RT concepts with NUIs that are mapped to their corresponding EPC classes via different role relationships. 154 NDF-RT concepts were mapped to EPC classes via the role relationship “has_Chemical_Structure”, 70 concepts via “has_PE” and 7 concepts via “has_MoA”. The coverage of NDF-RT and SPL for each category are calculated and shown in Table 2. Here, the coverage for NDF-RT is calculated by the number of NDF-RT concepts in each category divided by the total number of NDF-RT concepts (47,075 in total), and the coverage for SPL was calculated by the number of SPL labels in each category divided by the total number of SPL labels (36,568 in total). There are totally 497 EPC classes identified from the NDF-RT RDF repository, indicating that 71.2% (354 out of 497) EPC classes have been integrated into the EPC Indexing SPL labels.

**Table 2:** Mapping results by EPC classes based on EPC Indexing SPL Labels

**Category**	**Num. of unique NUIs**	**NDF-RT Coverage by EPC**	**Num. of unique setIds**	**SPL Coverage by EPC**
EPC	354	0.8%	853	2.3%
Chemical/Ingredient	154	0.3%	342	0.9%
PE	70	0.2%	201	0.6%
MoA	7	0.01%	10	0.03%
Total	585	1.2%	858	2.4%

“Category” denotes NDF-RT categories extracted from EPC indexing SPL;

“NUI” denotes identifier of NDF-RT;

“setId” denotes identifier of SPL.

### Results from RxNorm vs. NDF-RT mappings

We executed SQL queries and extracted 41,343 unique RxNorm entries (RxCUI) with NUIs and preferred names from RxNorm and NDF-RT mappings. To make linkages between SPL and NDF-RT, we searched each given RxCUI for a set of setIds from RXNSAT table. Finally, of 9,053 unique NUIs with setId, 6,611 unique NUIs belonging to three NDF-RT categories - “VA Product”, “Chemical/Ingredient”, and “EPC” are associated with 35,094 unique SPL setIds. In this step, we utilized NDF-RT category information to profile SPL.

The mapping results from RxNorm and NDF-RT mappings are listed in Table 3. There are 4,880 unique NDF-RT concepts from the category “VA Products”, linking to 20,937 unique SPL labels; whereas there are 1,730 unique chemical ingredients linking to 34,788 SPL labels. Comparing with the above EPC class mapping, only one EPC class identified and linked to 14 SPL labels have been integrated into RxNorm. The coverage is calculated by the number of concepts within each category divided by 47,075 NDF-RT concepts / 36,568 SPL labels. 96% SPLs have been covered by the RxNorm and NDF-RT mappings, whereas only 14.0% NDF-RT concepts have been linked to the SPL labels.

**Table 3:** Mapping results by RxNorm/NDF-RT mappings

**Category**	**Num. of unique NUIs**	**Coverage**	**Num. of unique setIds**	**Coverage**
VA Product	4,880	10.4%	20,937	57.3%
Chemical/Ingredient	1,730	3.7%	34,788	95.1%
EPC	1	0.002%	14	0.04%
Total	6,611	14.0%	35,094	96.0%

“Category”: NDF-RT categories from NDF-RT/RxNorm mappings;

“NUI”: identifier of NDF-RT;

“setId”: identifier of SPL.

Total 20,631 SPL labels have been mapped to more than one category. As an illustration, one SPL label “Cavan-EC SOD DHA” (setId = “0f2053f1-fd94-4a4a-b803-bca391d9e032”) has been mapped to “Fatty Acids, Omega-3 [Chemical/Ingredient]” (NUI = “N0000006244”), “SILICON DIOXIDE [VA Product]” (NUI = “N000158434”) and “Omega-3 Fatty Acid [EPC]” (NUI = “N0000175583”). In the meantime, there are 2,442 unique NUIs without category information, but they are duplicated with other concepts from the three categories. For example, “Mesna” (NUI=“N0000147595”) without category information has child concepts “MESNA 100MG/ML INJ” (NUI=“N0000156948”) assigned to “VA Product”. For this case, we did not count “Mesna” as a “VA Product”. However, we understand that “Mesna” is a drug class used to categorize its subclasses informed by “VA Product”.

### Results from RxNorm vs. SPL mappings

We first identified 35,480 unique SPL entries from RxNorm and SPL mappings with “SAB= MTHSPL”. And then we identified 15,615 unique RxCUIs that correspond to the SPL setIds by searching RXNSAT MySQL table. We used the term types “TTY” to classify SPL labels into ten categories. Each category with the number of unique RxCUIs, unique setIds and their coverage has been listed in the Table 4. Comparing with the 36,568 SPL labels from the NLM DailyMed and 965,968 concepts from RxNorm, 97.0% SPLs have been covered by RxNorm and SPL mappings, whereas only 1.6% RxNorm has been linked to the SPL labels.

**Table 4:** Mapping results from RxNorm and SPL mappings

**TTY**	**Num. of unique RxCUIs**	**Coverage by RxNorm**	**Num. of unique setIds**	**Coverage by SPL**
SBD	6714	0.70%	7087	19.38%
SCD	5981	0.6%	21127	21.9%
IN	1,834	0.2%	34,038	93.1%
PIN	773	0.08%	18,498	50.6%
BPCK	261	0.03%	259	0.7%
GPCK	48	0.005%	150	0.04%
BN	2	0.0002%	5	0.01%
MIN	2	0.0002%	3	0.008%
SY*	11,489	1.2%	21,438	58.6%
TMSY*	1,810	0.2%	4,208	11.5%
Total	15,615	1.6%	35,480	97.0%

“TTY” denotes term types from RxNorm;

“RxCUI” denotes identifier of RxNorm;

“setId” denotes identifier of SPL.

“*” denotes the concepts with SY and TMSY have overlaps with the concepts in other TTYs.

It is worthy to note that there are overlaps among concepts with different TTYs, such as SY and TMSY that denote synonyms of another TTY, thus concepts with SY or TMSY are overlapping with concepts with other TTYs. Also the same as RxNorm and NDF-RT mappings, there are overlaps for SPL labels among all of the categories.

### Network visualization

**Figure 8:**
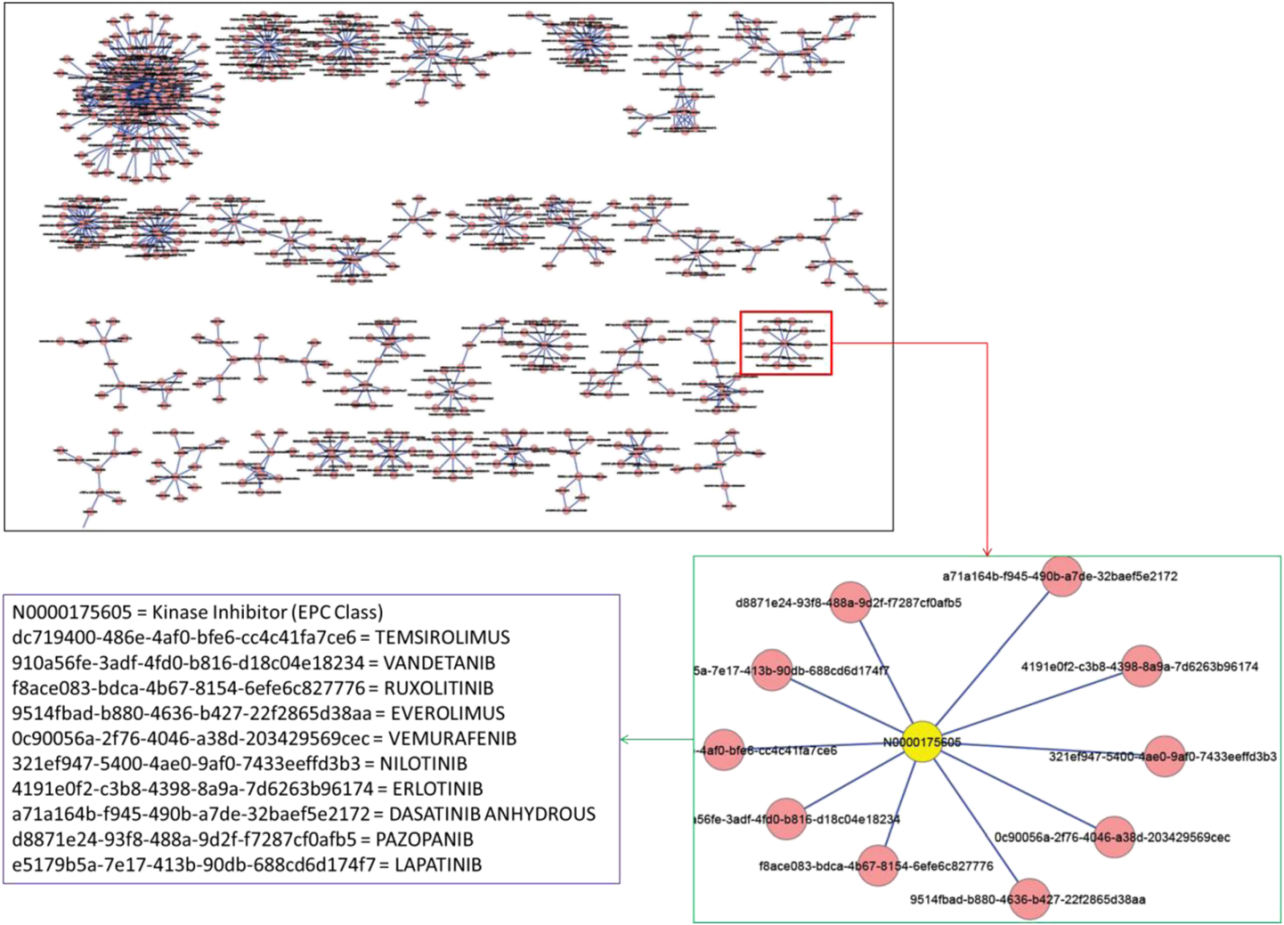
Network visualization of a drug and drug class network based on the profiling results of SPL drug labels (The upper left is the subsets of entire drug network built using the results from this study; the lower right is an enlarged subset of the network; the lower left illustrates the details about the sub-network shown in lower right).

The profiling results of SPL drug labels using RxNorm and NDF-RT not only demonstrate the connections among these three resources, but also help establish a drug/drug class network based on them. Within this network, the target and source nodes represent the concepts from SPLs, RxNorm or NDF-RT; the edges represent the category information. We are exploring Cytoscape [20] as a visualization tool to display and analyze the network. Figure 8 displays the network constructed using the results from this study. The upper right picture in Figure 8 shows a subset of the entire network; and the lower right picture shows one of the sub-networks with NDF-RT concepts as source nodes, SPL labels as target nodes, and category EPC as edge; and the details about nodes are shown in the lower left picture in Figure 8.

## Discussion

SPL labels contain a large portion of clinical drugs, and chemical/ingredients, as well as other possible drug categories. SPL labels as a very useful drug knowledge resource have been applied in clinical drug applications such as Adverse Drug Events (ADEs) detection from electronic medical records (EMRs). Notably, a number of studies are emerging recently to use the SPL labels for the purpose of drug safety surveillance. For example, in a project called SIDER, a public, computer-readable side effect resource that connects 888 drugs to 1450 side effect terms was developed using the SPL labels [21]. In a system called ADESSA, as another example, the ADEs were extracted from the SPL labels and mapped to the MedDRA terms and concepts, then utilized the UMLS to generate mappings between the MedDRA terms and the SNOMED CT concepts [22]. In a project at Mayo Clinic, the SPL labels were used in a framework for building a standardized ADE knowledge base known as ADEpedia [23] through combining ontology-based approaches with Semantic Web technology. In addition, Schadow conducted some other studies to evaluate the impact of SPL for medication knowledge management [24]. And Schadow also had successfully aligned SPL with associated terminologies to make drug-intolerance (allergy) decision support in computerized provider order entry (CPOE) systems in 2008 [25].

In this paper, we have successfully mapped SPL labels to NDF-RT and RxNorm, and categorized them using drug class information and clinical drug identification information respectively. 96.0% of SPL drug labels are mapped with NDF-RT categories whereas 97.0% of SPL drug labels are linked to RxNorm codes. The high SPL coverage by NDF-RT and RxNorm indicates that, on the one hand, the two drug ontologies are the appropriate data standards for normalizing drug information covered by SPL drug labels. On the other hand, the knowledge structure asserted in the two drug ontologies can be leveraged to analyze and enrich the SPL drug information.

As we mentioned above, RxNorm defines the term types to indicate the role an atom plays in its source (see Table 1) and specifies the relationships between the term types (see Figure 2). We utilized the term types to profile the SPL drug labels, which resulted in very useful insights on the characteristics of the existing SPL drug labels as an important drug knowledge source. We found that the majority of SPL drug labels (93.1%, see Table 4) are linked with the RxNorm term type “IN” which is defined as a compound or moiety that gives the drug its distinctive clinical properties. Only 21.9% of SPL drug labels are linked with the RxNorm term type “SCD” which is defined as the semantic clinical drugs with the combination of “Ingredient + Strength + Dose Form”. These findings indicate that when the SPL drug labels are utilized for a clinical drug application, the mappings through the ingredients would provide a better coverage (i.e., more sensitive) than through the combination of “Ingredient + Strength + Dose Form”. More importantly, the RxNorm term type “IN” is indirectly linked with the term type “SCD” through asserted relationships (see Figure 2). This provides additional options to optimizing the use of SPL drug labels in a clinical drug application through RxNorm.

Similarly, there is a content model asserted for modeling knowledge structure of NDF-RT (see Figure 1). We utilized the categorical drug class in the NDF-RT to classify the SPL drug labels. Our finding indicates that 95.1% of SPL drug labels are classified into the category “Chemical/Ingredient” and 57.3% of SPL drug labels are classified into the category “VA Product” which is analogous to the clinical drugs. While the finding from NDF-RT is consistent with what we have found in the RxNorm, NDF-RT provides more powerful analysis and aggregation capability because it contains a hierarchical concept structure that defines a rich set of domain-specific categories. For example, when a clinical application needs to collect drug information for all cardiovascular medications, NDF-RT provides a category “[CV000] CARDIOVASCULAR MEDICATIONS” which contains 16 direct subcategories and 1246 descendant classes in total (from NDF-RT version 2012.02.06). Using the asserted knowledge, the SPL drug labels that have been classified into the category “VA Product” will be able to aggregate to various domain-specific categories based on the requirements of a clinical application. This is also an important area we will explore in the future.

A drug and drug class network was built based on the SPL mappings with different data resources: EPC classes, NDF-RT and RxNorm. From such network, it can allow us to explore more drug class information for SPL drug labels and their connections with other clinical drugs. We are exploring Cytoscape [20] as a visualization tool to display and analyse the network. We consider that this is an important module of the system because it will provide a user friendly interface to allow end users to capture their target information efficiently. Note that we only integrated drug information into this network; we will integrate more drug / drug class resources, like PharmGKB [26], DrugBank [29], or National Drug Code (NDC) [27] into this network. In addition, the drug related phenotype information integration will be the next target in our future study. These integration efforts will make the network more useful to support clinical applications such as a clinical decision support system for clinicians.

Semantic Web technologies are being widely used in the biomedical research field. For instance, a well-known Linked Data application known as Bio2RDF [28] provides interlinked life science data to support biological knowledge discovery. It seamlessly integrates about fifty machine interpretable data to support federated queries for complex questions. LinkedSPLs [2], another SPL centred Linked Data resource, links SPL to DrugBank [29], ChEBI [30], etc., and makes both the original and extracted product label contents queriable using drug identifiers presented in these drug information resources. Although these Linked Data applications are focusing on data integration, none of them takes further steps to organize product labels from the perspective of drug class and clinical drug identification. In the present study, we applied EPC classes, NDF-RT and RxNorm to standardize and categorize SPL labels into different drug classes, aiming at integrating them into a standard drug / drug class network.

Semantic Web technology plays a key role in the implementation of our system. We represented the drug data from NDF-RT and SPL drug labels in RDF triples and hosted the RDF triples in a RDF triple store. We consider this makes the data integration, data management more feasible. In addition, running SPARQL queries against RDF store through SPARQL endpoint simplified our efforts on linking SPL drug labels to NDF-RT by using the predicates defined in the RDF triples. The NLM has not provided RDF based data format for RxNorm. In this study, we stored and analyzed the RxNorm data based on a relational database. In the future study, we will employ D2R [31] server for the RxNorm RDF transformation through defining mappings between the RRF-based relational database schema and a RDF data model, and also explore an RxNorm SPARQL endpoint offered recently by the NCBO BioPortal and integrate the RxNorm into the Semantic Web based framework of our system.

## Conclusions

In this study, we have successfully mapped SPL drug labels with RxNorm and NDF-RT. In total, 96.0% of SPL drug labels are mapped with NDF-RT categories whereas 97.0% of SPL drug labels are linked to RxNorm codes. We found that the majority of SPL drug labels are mapped to chemical ingredient concepts in both drug ontologies whereas a relatively small portion of SPL drug labels are mapped to clinical drug concepts. We believe the profiling outcomes produced by the study would provide useful insights on meaningful use of FDA SPL drug labels in clinical applications through standard drug ontologies such as NDF-RT and RxNorm.

We will continue the following investigations in the future. 1) Since existing SPL drug labels have been classified into a number of categories, including over-the-counter (OTC), prescription, animal, and so on, we will explore to integrate the information into the current drug network; 2) we will build a backbone drug network based on NDF-RT and integrate the network with more drug resources such as DrugBank, NDC, PharmGKB, etc.; 3) we will explore to build linkages between the drug/drug class and relevant phenotype/genotype.

## Competing interests

The authors declare that they have no conflict of interest.

## Authors’ contributions

GJ conceived the study; QZ carried out the data analysis; QZ and GJ wrote the paper; CGC reviewed the paper. All contributed to the intellectual evolution of this project. All authors have read and approved the final manuscript.
